# The significance of executive functioning in juvenile justice interventions: insights from clinical practice

**DOI:** 10.3389/fpsyg.2026.1809135

**Published:** 2026-04-13

**Authors:** Sterre L. van Haeringen, Annelinde R. E. Vandenbroucke, Hanna Swaab, Lucres M. C. Jansen

**Affiliations:** 1 Section Youth at Risk, Department of Child and Adolescent Psychiatry and Psychosocial Care, Amsterdam UMC, Vrije Universiteit Amsterdam, Amsterdam, Netherlands; 2 Mental Health, Amsterdam Public Health, Amsterdam, Netherlands; 3 Clinical Neurodevelopmental Sciences, Leiden University, Leiden, Netherlands; 4 Leiden Institute of Brain and Cognition, Leiden University, Leiden, Netherlands

**Keywords:** clinical insights, executive functioning, forensic intervention, justice-involved youth, juvenile justice institutions, neuropsychology

## Abstract

**Objective:**

Juvenile justice institutions (JJIs) aim to reduce recidivism and promote resocialization by tailoring interventions to youths' risks and needs. Executive functioning (EF)—cognitive processing supporting adaptive, goal-directed behavior—is closely linked to behavioral regulation and risk for delinquency. Given its continued development into early adulthood, EF is a potential target for intervention. However, it is still unclear how EF is incorporated into forensic clinical practice. This study explored how EF, and neuropsychological explanations more broadly, currently contribute to clinical decision-making within JJIs.

**Method:**

This qualitative study explored the significance of EF in clinical decision-making within JJIs. We conducted semi-structured, in-depth interviews with 13 coordinating clinicians from four Dutch JJIs. Interviews were analyzed using thematic analysis.

**Results:**

Four themes described the current role of EF in JJI practice: (1) general awareness and knowledge of EF, (2) the significance of EF in intervention planning, (3) the use of information on EF during intervention, and (4) challenges and opportunities for further integrating knowledge about EF in interventions. Across themes, EF, and neuropsychology in general, played a limited role. EF was typically considered implicitly, due to dominance of social-behavioral explanatory models of behavior and limited available information on individual youth's EF.

**Conclusion:**

Findings indicate that the contribution of EF in juvenile forensic practice regarding assessment, daily support and intervention is limited and largely implicit. By highlighting challenges and opportunities for integrating neuropsychological perspectives in daily juvenile forensic practice, this study provides valuable insights for translating neuropsychological knowledge into forensic youth care.

## 1 Introduction

The main priority of Dutch Juvenile Justice Institutions (JJIs)—apart from providing retribution and protection of society—is to resocialize justice-involved youth in order to prevent recidivism (Bruning et al., 2011). As a means to achieve this goal, alongside the provision of a pedagogical climate, a wide range of interventions (e.g., cognitive behavioral therapy, psychomotor therapy, systemic therapy) is offered to address the needs and risks of justice-involved youth. Despite these efforts, recidivism rates after residing within a JJI remain high at about 54% (Kessels, 2023). This leads to questions whether interventions are effectively targeting the specific developmental needs of individual youths. Whilst delinquent conduct can present itself in a similar manner, the driving mechanisms of this rule-breaking behavior differ for each individual. Incorporating neurocognitive mechanisms underlying rule-breaking behavior in combination with environmental factors, may increase the effectiveness of enhancing prosocial behavior through forensic interventions by shifting the focus from behavioral symptoms to the underlying cognitive processes driving individuals' actions (Jansen, 2022; Blair et al., 2025). An important neuropsychological factor to consider in forensic youth interventions is executive functioning (EF), which refers to a set of cognitive processes that support goal-directed, adaptive behavior, particularly in complex, dynamic, or socially demanding contexts (Diamond, 2013). In this paper, we will explore in which manner EF, and neuropsychological explanations more broadly, currently contribute to decision-making regarding the support and intervention targets in JJIs in the Netherlands.

To ensure that an intervention fits with an individual's risks and needs, it is important to assess how different personal characteristics influence not only behavior, but also the intervention effectiveness in preventing recidivism and promoting resocialization. This aligns with the risk need responsivity (RNR) model, which is based on assessing individual risk levels, criminogenic needs, and treatment responsivity (Bonta and Andrews, 2007, 2016). In light of individual risk levels and treatment responsivity, the role of neuropsychological processes underlying delinquent conduct has recently received more attention (Newsome and Cullen, 2017; Cheng et al., 2019; Jansen, 2022; Blair, 2024; van Horn et al., 2024).

A central construct within neuropsychology is EF, which plays a critical role in regulating socially adequate behavior in everyday life (Barkley, 2001; Beauchamp and Anderson, 2010). Executive functions are high-level cognitive functions that, by exerting influence over lower-level cognitive functions, influence a person's thinking and behavior through levels of cognitive control (Miller and Cohen, 2001; Barkley, 2012; Friedman and Miyake, 2017). Although the exact definition of EF is still debated (Jurado and Rosselli, 2007), it is widely accepted that it consists of three distinctive fundamental components: *inhibition*—the ability to suppress impulsive thoughts and behaviors, *working memory*—the ability to maintain and manipulate relevant information, and *cognitive flexibility*—the ability to adjust behavior in response to changing demands (e.g., Miyake et al., 2000; Lezak et al., 2012). Additionally, attentional control is considered to be foundational for effective EF development, as it plays a directive role in selecting and sustaining attention toward relevant social information (Posner and Rothbart, 2007). More complex cognitive functions such as planning and problem-solving build upon these core components. By directing how perceptions, thoughts, and emotions are processed, EF is crucial for processing social information and facilitating socially appropriate, goal-directed behavior (Crick and Dodge, 1996; Diamond, 2013). As prior studies have shown that EF is malleable, particularly during adolescence when the brain is still undergoing critical developmental changes, it is important to take into account what role EF plays in assessing the needs and risks of an individual during forensic intervention (Rice et al., 2023).

EF matures from childhood throughout late adolescence in a non-linear manner, with a significant spurt of maturation during adolescence and different developmental trajectories for individual executive functions (Huizinga et al., 2006; Best and Miller, 2010; Boelema et al., 2014; Tervo-Clemmens et al., 2023). This is linked to the ongoing developmental maturation of the prefrontal cognitive control systems, which typically reaches maximum capacity around 25 years of age (Luna et al., 2010). Whilst the development of EF is a trajectory that spans from prenatal stages to late adolescence, the first developmental periods in adolescence (pre- and mid adolescence, respectively, ages 10–15 and 15–18) are important sensitive periods for reaching the highest potential (Larsen and Luna, 2018). Besides genetic predisposition, exposure to environmental adversities, for example limited developmental support and education, can delay and disrupt EF development. When left unaccounted for, this can ultimately lead to executive dysfunctioning (Herrero-Roldán and Martín-Rodríguez, 2025). These deficits are often reflected in everyday difficulties with impulse control and adaptation to social demands. In turn, delays and deficits in EF are highly correlated with psychopathologies characterized by cognitive and behavioral impulsivity (e.g., attention deficit hyperactivity disorder, conduct disorder, autism spectrum disorder, psychopathy), as well as to delinquent and rule-breaking behaviors (Ogilvie et al., 2011; Schiffer et al., 2014; Hawes et al., 2016; Griffith et al., 2024; Jansen and Franse, 2024). Importantly, these associations are not specific for forensic populations: EF plays a central role in daily emotion and behavior regulation for all individuals.

Various studies based on population samples as well as justice-involved groups found that the extent of executive dysfunctioning varies related to the type of delinquent behavior that is displayed (i.e., violent vs. nonviolent, reactive vs. proactive) (Granvald and Marciszko, 2016; Kalvin and Bierman, 2017; Meijers et al., 2017; Hecht and Latzman, 2018). At the same time, similar rule-breaking behavior can be associated with different underlying executive dysfunctioning (Blair, 2024). Additionally, some studies have found that within a justice-involved population, those with serious executive dysfunctioning were more likely to reoffend (Seruca and Silva, 2015; Miura and Fuchigami, 2017). Of particular relevance is that EF should not solely be considered as a risk factor, but also as a protective factor: strong EF has a protective effect against criminal arrest (Nikulina and Widom, 2019).

The literature exploring the influence of EF on the efficacy of interventions for justice-involved youth is still in its infancy. In adult justice-involved populations, studies have shown that individual EF capacities are predictive of intervention dropout and success (Fishbein et al., 2009; Cornet et al., 2014). Targeting executive functions before or alongside intervention for behavioral change might therefore increase intervention success (Harvey et al., 2014). In a recent pilot study, Romero-Martínez et al. (2022) found that adult offenders of intimate partner violence who followed sessions of cognitive training focusing on improving various executive functions (e.g., sustained attention, working memory, problem-solving) on top of the standard behavioral intervention program, had lower levels of recidivism risk than those who followed the standard intervention program and a placebo cognitive training. In another pilot study with adolescents with severe aggression problems, Schippers et al. (2020) found that after following a training strongly focusing on improving EF, various executive functions improved and risk of violent recidivism was reduced. These first results suggest that improving EF could improve the success of forensic interventions.

As individual EF development is expected to influence treatment responsivity, it is important to look at the current contribution of knowledge about EF capabilities in decision-making regarding support and interventions for youth in forensic settings. During everyday practice, clinicians have to integrate knowledge across multiple aspects of functioning, as the difficulties justice-involved youth face are often multifaceted and not confined to one area (Hillege et al., 2018). In daily clinical practice, intervention decisions are often informed by clinicians' tacit knowledge, developed through training and clinical experience (Stewart and Chambless, 2007; Kazdin, 2008). In a recent study, Mathews et al. (2025) explored the added value of neuropsychological assessment reports in youth justice, and found that they increase forensic professionals' understanding of cognitive strengths and weaknesses underlying antisocial behavior. Therefore, this study aims to explore to what extent and in which manner knowledge about executive functions contributes to decision-making for support and intervention in JJIs in the Netherlands.

Semi-structured interviews with coordinating clinicians are used to reveal the range of considerations that are involved in planning interventions in JJIs concerning EF in daily support and intervention. Four questions are central: (1) to what extent are awareness and understanding of EF reflected in everyday support and intervention within JJIs? (2) In what way does information about a youth's EF influence decisions regarding intervention planning? (3) How does knowledge of EF influence the way in which interventions are executed? and (4) Which factors influence the integration of EF into interventions in JJIs? By addressing these questions, this study aims to contribute to a clearer understanding of the significance of EF in choosing the targets and methods of intervention within JJIs.

## 2 Methods

### 2.1 Design

The study performed semi-structured interviews with coordinating clinicians to explore in what ways executive functioning (EF) is considered in clinical decision-making regarding intervention plans and execution of interventions for justice-involved youth in juvenile justice institutions (JJIs) in the Netherlands. Interviews were conducted between December 2024 and June 2025 across four JJIs. Although the JJIs were familiar with the research team from a different ongoing project, participants did not have a personal relationship with the interviewer (SH), and SH had no personal or work relationships within the JJIs. The study was preregistered on the Open Science Framework,^1^ and ethical approval was granted by the Ethics Review Committee of the Institute of Education and Child Studies of the Leiden University (ECPW-2024/433).

### 2.2 Participants and recruitment

A total of 13 coordinating clinicians participated in the study from four different JJIs in the Netherlands. Coordinating clinicians are psychologists or remedial educationalists responsible for developing individual intervention plans, monitoring progress, and coordinating care for a residential unit within the JJI. Additionally, coordinating clinicians provide individual treatment for youths from other residential units in the institution. Participants were eligible if they were involved in both planning and executing intervention, were fluent in Dutch, and had at least 1 year of experience as a coordinating clinician within a JJI.

Recruitment was conducted in person or through email invitations in which all coordinating clinicians received a written overview of the study aims. Participants were recruited through convenience sampling: all coordinating clinicians were contacted, and those who responded and fit the inclusion criteria were included on a first come, first-served basis until saturation occurred. No one explicitly declined participation. Participant recruitment, selection, and data collection were carried out concurrently across all four sites. This resulted in a final sample of 13 participants; three JJIs with three participants each and one JJI with four participants. There are approximately 10 coordinating clinicians per JJI, meaning we included around 33% of the study population. Given the homogeneity of the target population, this number of participants for reaching saturation was expected (Guest et al., 2006). Saturation was operationalized as the point at which no new codes or concepts emerged (Fusch and Ness, 2015). All participants provided written informed consent, including explicit permission for audio recording, and received a small token of appreciation for their participation (a box of chocolates).

Of the 13 participants, 10 were female and three were male, with ages ranging from 30 to 47 years (*M* = 35.6, SD = 4.9) and professional experience working as a coordinating clinician ranging from 2 to 11 years (*M* = 5.9, SD = 2.7). Eight participants were legally certified healthcare psychologists (*GZ-psychologist, certified after 2 years of post-master professional training*), and five were master-level psychologists or remedial educationalists. The participants worked in different JJI contexts: regular short- and long-stay (more than 3 months), youths with mild intellectual disability, or youths in special care units. Participants provided a range of different individual interventions, including cognitive behavioral therapy (11/13 participants), schema therapy (8/13), systemic therapy (1/13), trauma-focused therapy (6/13), and aggression regulation therapy (4/13).

### 2.3 Data collection

Face-to-face interviews were conducted at the participants' workplace in a private setting to ensure confidentiality. No third parties were present during the interviews. Interviews lasted 39 to 57 min (*M* = 49, SD = 6) and were conducted by the trained first author (SH) using a semi-structured interview guide. This format allowed for both focused exploration of EF and flexibility to probe emerging themes of individual experiences of coordinating clinicians (Adams, 2015). At the outset of each interview, SH introduced herself by her areas of research and affiliations. Reflexive notes were written immediately after each interview to document initial impressions.

The interview began with questions about participants' daily responsibilities, followed by four main themes tied to the four research questions: (1) clinicians' general knowledge and views about neuropsychology and EF, (2) clinicians' use of information regarding EF in decisions on intervention planning, (3) clinicians' use of EF knowledge during the execution of interventions, and (4) challenges and facilitating factors in integrating EF into everyday practice. The interview guide is included in Supplementary material 1.

All interviews were audio recorded using an Olympus Digital Voice Recorder (VN-713PC). Recordings were transcribed verbatim by the first author and three trained research interns. All information that could be directly traced to the participant was omitted from the transcript. To ensure the reliability of the collected data, a form of member-checking was used (Goldblatt et al., 2011). Participants were sent the transcript of their interview and asked to review whether there were any factual inaccuracies in the transcript; for example, whether the words they used corresponded to their intended meanings (Varpio et al., 2017). If they believed there were factual inaccuracies in the transcript, they contacted the first author (SH). Participants generally brought forth minor grammatical errors, and no factual inaccuracies. Participants were not asked to comment on the thematic findings. After the transcripts were reviewed by the participants, analysis started.

### 2.4 Data analysis

The transcripts were imported into Atlas.ti (v.25) (Scientific Software Development GmbH, Berlin, Germany), a qualitative data analysis software package. The data was analyzed through a thematic analysis approach (Braun and Clarke, 2006; Naeem et al., 2023), adopting a primarily inductive and iterative process. In the first stage of coding, five interviews were read in-depth and openly coded by the first author (SH) and a master student. The master student received training in qualitative coding and was supervised throughout the process. Codes were compared and discussed collaboratively, with analytic memos used to document decision-making and refine the developing codebook. The coding structure was then reviewed and iteratively clustered into candidate themes and subthemes through ongoing comparison across the dataset with the entire research team. To strengthen the analytic process, the first and second author (SH, AV) independently coded two interviews and engaged in discussions to resolve differences in interpretation. This stage focused on reflexive dialogue, by means of discussing divergent interpretations, clarifying code definitions, and strengthen the coherence of the coding frame. The remaining six interviews were then coded by the first author. Only minor refinements of the coding structure occurred, supporting the adequacy and stability of the coding structure.

All quotes that are used in this manuscript were translated from Dutch by the use of ChatGPT v.4o and the translation prompt from the Maastricht University AI Prompt Library (Maastricht University, 2024). The translation was rigorously checked by SH for retainment of the verbatim character of the original statements.

### 2.5 Researcher reflexivity

Throughout data collection and analysis, the research team engaged in continuous reflection on their positionality. The authors are all Dutch, female, white, hold advanced academic degrees, and have disciplinary backgrounds in (neuro-)psychology, criminology, child and adolescent psychiatry, pedagogy, and cognitive neuroscience. The research team included a legally certified health care professional, and researchers with previous experience in conducting qualitative interview research in forensic settings. This interdisciplinary perspective facilitated a comprehensive and reflexive interpretation of the data, while efforts were made to avoid imposing a one-dimensional view of EF in the forensic context.

## 3 Results

The results obtained from the 13 semi-structured interviews are outlined along the lines of the four leading themes: (1) general awareness and understanding of EF, (2) role of EF in intervention plans, (3) use of EF knowledge during intervention execution, and (4) challenges and opportunities for integrating EF in daily practice. An overview of the subthemes in each theme can be found in Table 1. Coordinating clinicians expressed similar perspectives irrespective of their affiliated juvenile justice institution (JJI).

**Table 1:** Overview of themes and subthemes regarding the use of executive functioning.

Themes	Subthemes	Subthemes examples
General awareness and understanding	Dormant expertise	“*I don't recall much of that anymore…”* [P8]
	Generalized expertise	“*And that's where the challenge lies: having sufficient knowledge of all the sub-areas. Because in that sense, we really are generalists.”* [P6]
	Implicit awareness	“*But I do think that it's* [EF] *often more or less taken into account.”* [P3]
Intervention planning	Limited information available	“*I come across it* [EF] *every now and then, but for the most part, I don't*.” [P13]
	Implicitly considered for setting intervention targets	“[EF] *is more underlying…”* [P12]
	Reflection on necessity	“*I think it* [EF] *can add something extra.”* [P10]
Executing individual interventions	Adaptations due to lack of motivation or inability	“*And why you get stuck, well, whether it's inability or unwillingness, I can't figure that out.”* [P5]
	Development of EF in JJI	“*I think the system partly takes over those kinds of things* [EF skills] *for them.”* [P1]
Integration challenges and opportunities	Other explanatory models of behavior dominant	“*Because other themes are more at the forefront.”* [P6]
	Blind spot	“*I think we underestimate how often it* [EF] *actually plays a role.”* [P5]
	Abstract construct	“*It* [EF] *are really abstract terms for most people.”* [P9]
	Make added value of EF concrete	“*Then it* [EF] *has to offer new insights and tools for daily work.”* [P9]
	Explicate what is already implicitly done	“*It's not a matter of more integration, but making it* [EF] *more conscious.”* [P3]

EF, executive functioning; JJI, juvenile justice institutions.

### 3.1 General awareness and understanding

Based on participants' responses to questions about the use of EF and neuropsychological explanations more broadly in understanding youths' behavior, three subthemes emerged that explain the perceived significance of EF in day-to-day practice in JJIs.

#### 3.1.1 Dormant expertise

Eleven participants expressed difficulties defining the concept of neuropsychology and, albeit in a lesser extent, EF. Although all participants were able to associate EF with relevant constructs (mainly inhibition, attention, and planning), six participants reported feeling uncertain about their level of knowledge on the topic, stating that they did not know how to best describe EF:

“*I don't recall much of that anymore, do you realize how long ago that was?”* [P8]

In addition, this uncertainty was reflected in the way EF was often conflated with IQ, or primarily discussed in the context of clinical diagnoses such as ADHD, autism spectrum disorder, traumatic brain injury, or fetal alcohol syndrome. This was also shown in the way participants viewed EF as a relatively static trait, with limited room for development or intervention:

*But we see those* [executive functions] *as fairly static too. Like, this is the highest level that can be reached. You can teach some things in terms of behavior, but really, cognitive ability is usually just up to a certain level, and beyond that it just doesn't work*. [P3]

#### 3.1.2 Generalized explanatory approach of behavior

Participants describe that within their work, youths' behavior is often interpreted through explanatory models grounded in personality pathology, frequently attributed to trauma and attachment problems. Behavioral observations and prior forensic assessments form the basis for understanding rule-breaking behavior to ultimately reduce recidivism risk, in which a neuropsychological lens is not often applied. Given the heterogeneity of the justice-involved youth population who present a wide variety of problems, participants noted that their work is generally not directed toward identifying specific underlying processes driving particular behaviors:

“*So I think we often talk about what we see above the surface, but what lies beneath, we usually don't discuss*.” [P9]

Instead, they described using a more generalized approach that addresses multiple potential contributing factors simultaneously:

*Our main goal is simply to reduce risks, to lower the risk of reoffending. So, like I said, during a psychomedical case review, it's not like we literally say ‘okay, how is he functioning from a neurobiological perspective, or what about attachment?'. It doesn't get addressed that concretely, though I really do think it's taken into account. But it's not really separated from other factors*. [P4]

#### 3.1.3 Implicitly aware

Even though alternative explanatory models of behavior dominate, 11 participants believed that EF is implicitly incorporated in everyday practice. One participant says:

“*I think we're always considering it, just not consciously*.” [P10]

Four participants noted that elements of EF are indirectly addressed through items in risk assessments tools, such as coping, problem-solving and self-sufficiency. While four participants indicated that some aspects of EF were explicitly embedded in their work, though not all, seven participants reported that EF was incorporated only implicitly, recognizing it in youths' behavior without labeling it with specific terms (e.g., inhibition, working memory). Instead, they tended to discuss these behaviors in more general, non-jargon language:

*It's also not our primary focus. Executive functions aren't considered a risk factor, for example. […] Of course, it's something we do take into account when looking at self-sufficiency and, controlling behavior is definitely very important. We also consider impulsivity. And of course intelligence is something we take into account too, but it's not something we specifically focus on*. [P12]

### 3.2 Executive functioning in intervention planning

Participants responses on the consideration of EF during intervention planning lead to three overarching subthemes that portray the current significance of EF during this decision-making process.

#### 3.2.1 Limited information available

Participants describe that the readily available information from forensic assessment reports, behavioral observations done by residential staff, and offense analysis generally provided sufficient insight into which intervention and aims should be proposed for each youth. Consequently, additional assessments are rarely conducted, except in a few cases each year for diagnostic clarification or IQ-testing. Therefore, participants expressed that for EF to be considered explicitly, it must be referenced in the existing documentation, most likely within the forensic assessment report. However, participants noted that explicit references to EF are uncommon:

“*I come across it every now and then, but for the most part, I don't*.” [P13]

Another participant further reflected that the perceived absence of the mentioning of EF might also stem from clinicians not recognizing it when present:

*Because I think that if we read a forensic assessment report in a different way, we might secretly be able to take out more neurobiological or neuropsychological information than we do now. Sometimes those tasks might not have been administered, but I think that in a forensic assessment report, especially in a double report, it's often still there. It's just that we might not recognize it very well from the reports at the moment*. [P2]

#### 3.2.2 Implicitly considered during intervention targets discussions

Six participants reported that EF is sometimes explicitly addressed during psychomedical case reviews. These instances typically occurred only when executive functions were mentioned in the forensic assessment report or when an expert on the topic was present; circumstances the participants described as infrequent:

“*So, of course, that also depends somewhat on who is present and what expertise they bring*.” [P2]

More often, the role of EF is implicit. All participants expressed that it is considered during discussions on whether the cognitive demands of an intervention would exceed a youth's capacity:

[…] *if someone has higher cognitive functioning, you can also use somewhat more complex therapies*. [P7].

Similarly, in formulating intervention aims, strengthening EF is never an explicit goal, but almost always an unintended positive side effect:

*But I don't think my primary focus in therapy is to expand cognitive skills. I do think it's often an unintended side effect, or, well, a nice bonus. But it's not the main focus of my therapy*. [P1]

#### 3.2.3 Reflection on necessity

During the interview, participants often reflected on whether EF should be more explicitly incorporated into intervention decision-making. On this matter, participants were divided. Some doubted it's added value, believing it would only be relevant for a small subset of youths and therefore too narrow a focus:

“*I think that's so specific, that it wouldn't cover enough for us* […]” [P8]

Others felt it could be beneficial, particularly for understanding the underlying processes of problematic behavior, and thereby helping to clarify what can reasonably be expected from a youth during intervention:

*But if every coordinating clinician really knows what executive functions are, they'll notice issues with it more quickly. And then, when you actually start with treatment, you know what the core problems are, and you can see what a youth is struggling with*. [P10]

### 3.3 Executive functioning during individual intervention execution

Participants emphasized that tailoring interventions to the diverse risks and needs of justice-involved youth is essential for treatment effectiveness. Two subthemes emerged when participants were asked about the current significance of EF during intervention execution and the broader pedagogical climate of the JJI.

#### 3.3.1 Alterations because of motivation or inability

During interventions, seven participants observe that youths sometimes experience cognitive overload. To adapt the intervention to the cognitive level of the youth, all participants report making adjustments, such as repeating exercises more often, shortening sessions, using visual aids, simplifying language, and modifying homework assignments. Similar adjustment instructions are given to residential staff in their daily support for youth. However, participants expressed that because EF is rarely made explicit in daily practice, these adjustments are often attributed to account for a lack of motivation rather than to executive function deficits:

“[…] *for example, if he doesn't do his homework or it isn't done well, I wouldn't immediately attribute that to executive functions. I'd see it more as a matter of motivation*.” [P11].

This is reflected in the difficulty ten participants experience in distinguishing whether a youth's resistance to interventions arises from a lack of motivation or from limited cognitive capacities:

“*And why you get stuck, well, whether it's inability or unwillingness, I can't figure that out*.” [P5]

All participants nevertheless noted that interventions depend on cognitive abilities, meaning that these abilities must be engaged for the intervention to be effective. Consequently, when EF is underdeveloped or impaired, this can reduce the intervention's impact.

#### 3.3.2 Development of EF in JJI

When asked about potential growth in EF during stay in a JJI, participants generally found it difficult to determine how such development occurred, as it was described to never be explicitly targeted during intervention. While all expected that EF might improve as an unintended side effect of interventions, they were uncertain how to track this growth or separate it from natural maturation. With respect to the contribution of the general pedagogical climate of the JJI, seven participants believed that the daily structure supported EF development by fostering skills and providing stability:

*I'm not sure if that's always the case, but I do think we help youth structure their day and teach them skills they may not have fully learned during their childhood*. [P9]

Nine participants, however, also felt that this highly regulated structure might hinder development, as youth are not challenged to take responsibility for their own daily activities:

*I think that here, the whole day is pre-structured. So they're not really challenged to practice planning skills or things like that on their own. Everything here is so regulated that I don't get the sense they actively learn those skills very much*. [P2]

### 3.4 Challenges and facilitators for integrating executive functioning in the JJI

As participants described the role of EF as primarily implicit in daily practice, five key challenges and facilitating factors were identified for integrating it as a more explicitly in day-to-day practice.

#### 3.4.1 Other explanatory models of behavior dominant

Participants emphasized that, given the heterogeneity of justice-involved youth, many different risks and needs must be considered. As a result, choices on what to prioritize are inevitable, and in current practice personality pathology, attachment issues, and trauma are often regarded as more informative than neuropsychological frameworks including EF:

“*I think there are many things* [to consider]*, and I don't think this one* [executive functioning] *is at the top of my list*.” [P6]

One participant, however, reflected that the emphasis on these factors may stem from habit, rather than certainty of their relative importance for understanding behavior:

*I do think attachment and the development of personality structure play a significant role. We make that role more important. I don't know—I wouldn't dare say it's more important for delinquent behavior and risks. I just don't have enough knowledge about that*. [P2]

#### 3.4.2 Blind spot

Participants often describe EF as a “blind spot,” meaning that it's not often considered or discussed explicitly when trying to understand behavior. Participants expressed that this “blind spot” reinforces the tendency to rely on other explanatory models of behavior, which in turn could sustain the neglect of the role of EF, creating a self-reinforcing bias for looking at behavior:

“*Well, because I think we often skip that perspective. Or we're not really aware of it. Or only occasionally aware.”* [P1].

#### 3.4.3 Abstract construct

Participants generally describe EF as an abstract concept that is difficult to link to concrete behavior. Using a neuropsychological model was seen as a risk to create confusion with both residential staff as youth:

“*I think I'd lose people then.”* [P9]

Ten participants also attributed the challenge to integrate EF to their own experienced limited expertise. Although all had received some education on the topic, many, including the healthcare psychologists (GZ-psychologists), felt they lacked the practical knowledge to apply this knowledge in everyday work:

“*I don't think we all have it at our fingertips*, [not like] ‘*oh yes, this is what executive functioning looks like, and we can look at it through that lens'.”* [P13].

As this expertise is often not readily accessible to coordinating clinicians, residential staff are also rarely instructed to recognize executive dysfunction in behavioral patterns:

[…] *because I have little knowledge about it* [executive functioning]*, or at least don't actively share it or explore it in depth, it doesn't really filter through me to the team*. [P2]

#### 3.4.4 Make added value of EF concrete

Participants were also asked what might facilitate a more explicit integration of EF in daily practice. Given that EF is often described as a “blind spot” in current practice, participants emphasized the importance of first making the concept concrete and recognizable. In particular, participants noted that, given the limited time and staff resources, as well as the many other behavioral factors that need to be considered, clearly articulating the added value of EF in understanding behavioral risks would increase support for incorporating this perspective more consistently into daily practice:

*Then I'd mainly just want to know what the added value is, compared to what we already do. I think that's the main point*. [P8].

To achieve this, participants pointed to the need for education and practical tools that explicitly link executive functions to observable behavior. Practical training could for instance consist of providing guidance on how to adapt interventions to challenges in executive functions:

*Well, I think it can be helpful when you have more insight into situations that occasionally arise, to what extent something originates from a youth's limited executive functions. How do you then take that into account in therapy or when designing an intervention plan? And what can you additionally do—beyond what you're already doing—to address it properly?* [P11]

Another participant suggested a simple manual with example treatment goals related to EF that coordinating clinicians could keep in mind:

“*I just think it should be made as accessible as possible, in the sense that there's a list of example treatment goals in this area.”* [P5].

In addition, participants suggested having a dedicated person to keep the topic of EF alive within the JJI. While it is unrealistic to expect all staff to become experts in EF, one clinician, for instance a clinical neuropsychologist, could take the lead in ensuring EF remains on the agenda.

#### 3.4.5 Explicate what is already implicitly done

Finally, eight participants underscored that EF is already often implicitly considered in daily practice, but that it often goes unnoticed. In their view, the solution may lie less in further integration of EF and more in making the implicit explicit:

*Well, I think a lot of it already happens unconsciously. Also in the care plans. So maybe it's not a matter of more integration, but making it more conscious. I think that would help, because if it happens unconsciously, you probably miss it more often*. [P3]

## 4 Discussion

This study explored to what extent and in which manner coordinating clinicians currently consider executive functioning (EF) when choosing the targets and methods of intervention within juvenile justice institutions (JJIs) in the Netherlands. The current role of EF in daily support and intervention practice was discussed with coordinating clinicians along four central themes: (1) general awareness and understanding of EF, (2) the role of EF in intervention planning, (3) the use of EF knowledge during intervention execution, and (4) challenges and opportunities for integrating EF in daily practice. This study focused on the significance of EF as a part of neuropsychological functioning. However, the results indicated that neuropsychological frameworks more broadly played a limited role in explaining rule-breaking behavior in juvenile forensic practice. EF, and neuropsychological functioning more broadly, were typically considered implicitly, due to dormant expertise on the topic, the common practice of using broader behavioral and psychosocial explanatory models, and limited availability of individual youth's EF-related information. As applying a neuropsychological lens was not common practice, this might explain why participants described EF as a “blind spot” or an abstract construct that was difficult to operationalize in everyday support practice. This sentiment was also reflected in how they thought the broader pedagogical climate and interventions within JJIs might influence EF development, as well as how individual differences in EF could affect interventions effectiveness. Given the largely implicit role of EF and neuropsychological perspectives in current JJI practice, participants reflected on both challenges and opportunities associated with making these frameworks more explicit.

This first main finding is that neuropsychological frameworks play a limited role in daily clinical practice in the JJIs. Coordinating clinicians are responsible for planning and executing interventions for justice-involved youth in order to reduce recidivism and promote resocialization. To do so, participants describe commonly using explanatory models of behavior grounded in trauma, attachment, and personality development to interpret youths' rule-breaking behavior, consistent with the pedagogical orientation of Dutch JJIs (Bruning et al., 2011). Identifying specific neuropsychological mechanisms that contribute to these behaviors is not typically part of routine practice. Consequently, participants describe that neuropsychological frameworks—and concepts related to EF in particular—are not explicitly incorporated into day-to-day clinical decision-making.

Emerging research suggests that integrating neuropsychological information in intervention planning and execution may benefit clinical practice (Allott et al., 2011; Watt and Crowe, 2018; Donders, 2020; Mathews et al., 2025). In our study as well, coordinating clinicians acknowledged the importance of accounting for youths' cognitive capacities specifically in order to avoid exceeding these during intervention. However, at the same time, some participants expressed reluctance to explicitly incorporate EF into intervention decision-making. This hesitation may stem from the current practice in which underlying neuropsychological processes are not often considered, which obscures cases when recognizing executive functions is pivotal. As such, the challenge may not be a lack of willingness, but rather the absence of concrete frameworks that can translate EF into clear, actionable guidance for forensic professionals that fit within the existing pedagogical approach in the JJI. Although research on how these frameworks can be integrated is sparse, the first steps toward ways of integration are being taken. For example, in a recent study, a neuropsychological report added to the case files of justice-involved youth facilitated clinicians with better insight in the strengths and weaknesses of individual youth (Mathews et al., 2025).

The second main finding concerns the implicit manner in which coordinating clinicians incorporate EF information in the absence of explicit frameworks for everyday practice. Due to the heterogeneity of problems among justice-involved youth, combined with substantial time and resource constraints, participants noted that they often rely on observable behavior. This aligns with prior studies illustrating that forensic clinicians often make decisions based on tacit, experience-based knowledge, developed through practical experience without referencing back to underlying theoretical constructs or theories (Stewart and Chambless, 2007; Kazdin, 2008). Our findings reflect this pattern: coordinating clinicians described adjusting interventions to youths' cognitive capacities, yet these adjustments are typically framed in general terms rather than through the lens of specific executive functions. Five underlying reasons might contribute to this and are outlined below.

First, this tendency to implicitly consider EF might be reinforced by the findings that participants typically don't encounter the use of EF in their daily work. For instance, participants infrequently encountered references to EF or neuropsychological constructs more broadly in the information available on youths' behavior in for instance assessment reports or observations done by residential staff. Participants expressed that because of their own lack of knowledge on EF, they are not able to support residential staff to recognize or assess the role of EF in youths' behavior. Therefore, residential staff may fail to identify behavioral patterns that could be indicative of executive dysfunction. As a result, the information the coordinating clinicians receive based on the observations done by the residential staff lacks the necessary depth to support the consideration of EF as a potential explanation for rule-breaking behavior. Notably, little research has been done in mapping if and in what way neuropsychological constructs including EF are part of juvenile assessment reports.

Second, the implicit use of EF may result from lack of a clear conceptual definition of EF amongst coordinating clinicians. Participants frequently used terms such as EF and intelligence interchangeably, suggesting the absence of a grounded working definition when discussing EF processes. There are, however, important differences between intelligence and EF that influence their relation to rule-breaking behavior: intelligence refers to the ability to learn, understand, and apply knowledge, whilst EF encompasses the cognitive processes needed for regulated, goal-directed behavior (Friedman et al., 2006). Moreover, intelligence is relatively stable during adolescence, whereas executive functions continue to develop (e.g., Huizinga et al., 2006). Thereby, the lack of a clear definition of EF risks missing developmental opportunities for targeted intervention.

Third, although participants described adjusting interventions to match youths' cognitive capacities, it was often unclear to the clinicians whether these adaptations responded to motivational difficulties or underlying EF deficits. Motivation is widely recognized as a key challenge in treating justice-involved youth, and it is a central construct of the responsivity domain in the RNR model (Bonta and Andrews, 2007, 2016). It is important to note that prior studies have highlighted the close link between EF and motivation: stronger executive functions support better self-regulation, which in turn facilitates motivation (Barkley, 2001; 2012). This suggests that training EF should have a positive effect on treatment motivation. In addition, when it is clear to clinicians whether a youth does not participate well in an intervention due to lack of motivation or underdeveloped EF, this can lead to more effective adaptations, as motivational issues or cognitive incapabilities might call for a different approach. Furthermore, when EF are more explicitly discussed with youth, it might also be used as a form of psychoeducation which can foster increased treatment motivation. Indeed, Mathews et al. (2025) found that when justice-involved youth received psychoeducation based on their neuropsychological assessments, they reported greater insight in their own strengths and weaknesses, which improved motivation.

Fourth, coordinating clinicians tend to prevail more explicitly visible explanatory factors for rule-breaking behavior, such as trauma or attachment. As a result, rule-breaking behavior may be interpreted mainly at a behavioral level, with limited attention to the underlying neuropsychological processes that contribute to it. This creates the risk that these processes remain unaddressed during intervention. Indeed, participants frequently described EF as a blind spot. Many participants expressed uncertainty about when EF plays an important role in understanding youth's behavior. A key reason for this might be that many participants perceive EF as a complex and abstract topic, with limited practical knowledge and implications available. This sentiment can be understood when looking at the plethora of possible neuropsychological assessments available, but with no clear cut recommendations being made which assessments best fit with their target group and what clinical consequences can be derived from it (Péron, 2024).

Fifth, participants reflected on how the juvenile justice environment itself may shape EF. Some expressed concerns about potential negative effects, echoing findings that prolonged stays in correctional settings can impair EF (Meijers et al., 2018; Umbach et al., 2018). At the same time, clinicians emphasized the pedagogical potential of JJIs to support the development of executive skills. Although research on strengthening EF to reduce delinquency is still in its infancy, recent studies demonstrate promising outcomes: interventions targeting EF can enhance cognitive performance, improve intervention success, and reduce recidivism (Harvey et al., 2014; Schippers et al., 2020; Romero-Martínez et al., 2022). Notably, even low-intensity approaches may be viable; a pilot study showed that unsupervised cognitive training during cell time improved neurocognitive functioning (Rowlands et al., 2020). Such findings highlight practical opportunities for incorporating EF more deliberately into daily JJI practice. Tailoring developmental support and interventions to both strengthen underdeveloped executive functions and harness executive functions that are already strong, could improve intervention efficacy and promote resocialization.

Finally, participants brought forth challenges and facilitating factors for integrating EF, and more broadly the neuropsychological framework, in day-to-day practice. As the coordinating clinicians mention the concept of EF to be ambiguous, they highlight the need for concrete guidelines and practical tools to support in more explicitly integrating EF into daily practice. Participants emphasized that linking EF to observable behavior would make them more likely to consider it systematically. While a one-size-fits manual is not feasible, promising first steps toward creating guidelines for assessment are being made. Hutten et al. (2024) identified a top three for neuropsychological tests that are suitable for capturing cognitive deficits linked to reactive and proactive aggression. Clarifying the value of EF beyond currently used frameworks for understanding delinquent behavior in forensic juvenile settings would also help coordinating clinicians appreciate its added relevance. Given the heterogeneity of the justice-involved youth population, it is unrealistic to expect coordinators to become experts in every domain. Consistent with participants' suggestions, appointing a dedicated spokesperson, supported by the directorate, may help ensure that EF remains on the agenda within JJIs.

### 4.1 Limitations

Despite its contributions, this study also has several limitations. First, as the primary aim was to describe how EF is currently considered during forensic youth interventions, other explanatory models of youth behavior were not examined. Consequently, this study cannot describe to what extent such models may be informed by neuropsychological knowledge in current forensic practice. Second, the study sample was limited to coordinating clinicians. Although they play a central role in interventions in a JJI, other professionals, such as residential staff, also have a significant influence on the development of justice-involved youths and the pedagogical climate in general. They interact with youth more frequently, provide direct guidance during daily interactions, and may therefore have different perspectives on how (or whether) EF is taken into account. Third, none of the participants provided practical therapies (e.g., music therapy, psychomotor therapy). Given that coordinating clinicians mention frequently recommending such interventions for youth with limited cognitive capacities, it could be that practitioners providing such therapies have different views or experience in considering EF during everyday practice.

### 4.2 Practical implications and future directions

The descriptive nature of this study precludes normative judgments about whether the mainly implicit use of knowledge on EF in clinical decision-making is detrimental or beneficial for preventing recidivism and promoting resocialization of justice-involved youth. Nevertheless, the results provide important insights into the decision-making processes that guide forensic youth interventions. This is a field that remains understudied and is hard to grasp, partly due to the substantial reliance on individual professional experience (Stewart and Chambless, 2007; Kazdin, 2008; Hillege et al., 2018). At a time when neuropsychological perspectives on youth delinquency are gaining momentum (Cheng et al., 2019; Jansen, 2022; Blair, 2024; Blair et al., 2025), our findings highlight the importance of translating scientific knowledge into formats that align with the needs of everyday forensic practice. Not as abstract constructs, but as practical tools to strengthen forensic interventions and resocialization outcomes. Therefore, the following recommendations are made for advancing forensic neuropsychological research and practice:

1) Accessible assessments: future studies should employ measures of EF (and related neuropsychological constructs) that are feasible to administer and straightforward to interpret for forensic professionals who may have limited neuropsychological expertise.
2) Translation: education is needed that explains how deficits in EF give rise to rule-breaking behavior through underlying neuropsychological mechanisms. By clearly demonstrating to clinicians that these EF-related problems are present in justice-involved youth, professionals are better equipped to recognize such difficulties in day-to-day practice.
3) Practice-oriented tools: guidelines for incorporating neuropsychological knowledge into the context providing interventions for justice-involved youth should be co-developed with forensic professionals to ensure practical relevance and utility.
4) Treatment effects: longitudinal studies should investigate how executive functions develop over the course of a stay in a JJI, with specific attention to how different interventions and pedagogical factors influence this development and to what extent efficacy of interventions may be depending on the stage of development of EF. Such studies could shed light on the importance of individual EF profiles and clarify what supports or hinders EF growth.

## 5 Conclusion

To our knowledge, this study is the first to describe how the neuropsychological construct of executive functioning (EF) is considered in clinical decision-making for justice-involved youth. By interviewing 13 coordinating clinicians from four JJIs in the Netherlands, this study contributes a representative perspective on daily practice, decision-making with respect to interventions, and intervention execution. By highlighting the implicit role EF plays in practice, the findings underscore both the challenges and opportunities of integrating neuropsychological perspectives into forensic youth care. Recommendations are made for bridging the gap between science and practice. Ensuring that neuropsychological knowledge is meaningfully translated into clinical decision-making may enhance the effectiveness of forensic interventions and ultimately support better developmental and resocialization outcomes for justice-involved youth.

## Data Availability

Due to ethical concerns and the sensitivity of the data, supporting data cannot be made openly available. Information about the data and conditions for access can be discussed with the corresponding author upon request.

## Footnotes

1
https://doi.org/10.17605/OSF.IO/R4KZX
