# Supplementary material for: The significance of executive functioning in juvenile justice interventions: insights from clinical practice
Source: Front Psychol. 2026 Apr 13;17:1809135. doi: 10.3389/fpsyg.2026.1809135 (PMC13110986; doi:10.3389/fpsyg.2026.1809135)
Supplement: Supplementary file 1 [file Supplementary_file_1.docx]

Supplementary Material 1. Interview guide

1. General background
   1. Role and responsibilities within the (R)JJI
   2. Work experience in forensic youth care, specifically in juvenile justice institutions
      1. Interventions they personally provide
   3. What do they think about when they hear the term ‘neuropsychology’?

Based on the answers given to the above question, further discussion will be directed toward executive functions if the participant mentions them. If executive functions are not mentioned, the conversation will be steered toward this topic.

1. General views on executive functioning
   1. Education and knowledge regarding executive functioning
   2. How do they define executive functioning
   3. In what way, in their view, does executive functioning play a role in adolescent development?
   4. How much importance does the JJI place on executive functioning?

Next, a brief background will be provided on how executive functions are defined in this study, highlighting the three core functions: inhibition, cognitive flexibility, and working memory. Each will be described in relation to its influence on behavior:

- Inhibition: the ability to suppress immediate impulses, such as not reacting instantly to an external stimulus.
- Cognitive flexibility: the ability to adapt behavior to changing situations, new information, or unexpected circumstances
- Working memory: the ability to temporarily hold and manipulate information, for example, remembering instructions.

How do these three (or other executive functions mentioned by the participant) play a role in adolescent development according to the coordinating clinician?

1. Executive functioning in the intervention decision-making process
   1. In which manner is executive functioning taken into account when determining the type of intervention?
      1. At which stages does it play a role?
      2. To what extent is it considered when determining intervention intensity?
   2. How is a youth’s executive functioning assessed?
      1. Which instrument, tests, or observations are used?
   3. Can they provide examples of how the results of an executive functioning assessment have led to specific intervention decisions?
2. Cognitive characteristics and intervention execution
   1. How, in their view, does a youth’s executive functioning affect the way interventions are implemented?
   2. Are adaptations made in intervention execution to account for executive functioning limitations?
      1. If yes, which adaptations?
   3. Is strengthening executive functions addressed as a standalone treatment goal?
      1. If yes, why and how?
3. Factors influencing the integration of executive functioning?
   1. Which organizational factors may influence the integration of executive functioning into decision-making and intervention execution?
   2. To what extent is there collaboration with other professionals (*e.g.,* psychiatrists, psychologists, educational psychologists) to integrate executive functioning into the intervention process?
   3. Are coordinating clinicians sufficiently trained and supported in recognizing and applying insights on executive functioning?
4. Experiences and challenges
   1. What challenges do they experience in integrating executive functioning into their decision-making and intervention execution?
   2. Can they identify successful strategies or approaches that help account for executive functioning?
   3. Is it necessary to better integrate executive functioning into the intervention decision-making process?
      1. If yes, what do they need to achieve this?
      2. Do they have suggestions for improving the current approach?
